# Characteristics and Demography of a Free-Ranging Ethiopian Hedgehog, *Paraechinus aethiopicus,* Population in Qatar

**DOI:** 10.3390/ani10060951

**Published:** 2020-05-30

**Authors:** Carly Pettett, David W. Macdonald, Afra Al-Hajiri, Hayat Al-Jabiry, Nobuyuki Yamaguchi

**Affiliations:** 1 Wildlife Conservation Research Unit, Department of Zoology, Recanati-Kaplan Centre, University of Oxford, Tubney House, Abingdon Road, Tubney, Oxfordshire OX13 5QL, UK; carly.pettett@gmail.com (C.P.); david.macdonald@zoo.ox.ac.uk (D.W.M.); 2 Department of Biological and Environmental Sciences, Qatar University, P.O. Box 2713 Doha, Qatar; xxafra@yahoo.com (A.A.-H.); haljabiry@qu.edu.qa (H.A.-J.); 3 Institute of Tropical Biodiversity and Sustainable Development, Universiti Malaysia Terengganu, Kuala Nerus 21030, Terengganu, Malaysia

**Keywords:** Arabia, arid environment, desert, Middle East, density, survival, capture, abundance, population dynamics, small mammal

## Abstract

**Simple Summary:**

Information on population characteristics of *Paraechinusis* is valuable for ensuring long term survival of populations, however, studies are currently lacking. Here we investigate the population dynamics of Ethiopian hedgehogs based on a capture study in Qatar by fitting several statistical models. Over the 19 months of the study, we estimate a mean population of 60 hedgehogs, giving a density of 7 hedgehogs per km^2^ in our 8.5 km^2^ search area. The monthly abundance of hedgehogs decreased over the study and although survival was constant over the study period, with a mean monthly rate of 75%, there was a decline in the number of new entrants over time. We also studied these parameters over one year, excluding winter, and found that monthly estimates of juvenile and subadult survival decreased over time. We surmise that survival of juveniles may be a factor in the decrease in abundance and there may be implications for the persistence of this population in the future, with human influenced resources playing an important role. We caught between 91.3% and 100% of the estimated population at this site, indicating that our capture methodology was efficient. We conclude that the methodology used here is transferrable to other hedgehog species.

**Abstract:**

Information on population characteristics of *Paraechinusis* is valuable for ensuring long term survival of populations, however, studies are currently lacking. Here we investigate the population dynamics of Ethiopian hedgehogs based on a capture-mark-recapture study in Qatar by fitting Jolly-Seber and Cormack-Jolly-Seber models. Over the 19 months of the study, we estimate a mean population of 60 hedgehogs, giving a density of 7 hedgehogs per km^2^ in our 8.5 km^2^ search area. The monthly abundance of hedgehogs decreased over the study and although survival was constant over the study period, with a mean monthly rate of 75%, there was a decline in the number of new entrants over time. We also studied these parameters over one year, excluding winter, and found that monthly estimates of juvenile and subadult survival decreased over time. We surmise that survival of juveniles may be a factor in the decrease in abundance and there may be implications for the persistence of this population, with anthropogenic influenced resources playing an important role. We caught between 91.3% and 100% of the estimated population at this site, indicating that our capture methodology was efficient. We conclude that the methodology used here is transferrable to other hedgehog species.

## 1. Introduction

Hedgehogs are small terrestrial mammals with a spiny integument in the subfamily Erinaceinae, of which 16 species in five genera are currently recognized [1,2]. Extensive research on the European hedgehog (*Erinaceus europaeus*) has led to the common notion that hedgehogs are characteristic of the moist temperate environments of the world. However, many hedgehog species occur in arid and semi-arid environments, such as the “desert hedgehogs” of the genus *Paraechinus*, and yet, little is known about their ecology and behavior in these arid environments [1,2,3,4,5,6,7,8,9,10]. More than 25 years ago, in his monograph of hedgehogs, Reeve (1994) [1] expressed his frustration by stating “There is a frustrating lack of further studies … in non-European hedgehogs … There is a clear need for much more fundamental work on all these and other, as yet unstudied, hedgehog species”. Sadly, although there has been some work on ecology, behavior, and physiology of non-European hedgehog species in the past 25 years [3,4,5,6,7,8,9,10,11,12,13], basic information about their population characteristics is still largely lacking.

The Ethiopian Hedgehog (*Paraechinus aethiopicus,* Ehrenberg, 1832), which is well-adapted to arid environments, has a wide distribution across North Africa and the Middle East, including the Arabian Peninsula [1,2], and is the only native hedgehog species in Qatar [14]. There has been some recent study on the habitat use and home range of the species [6,13], as well as on the timing of breeding [5,9], hibernation [3,8,9], and behavior in winter [11]. However, there are no previous studies on the population density and dynamics of *Paraechinus* hedgehogs.

There are several sampling methods that are applicable to hedgehogs that have previously been used to investigate local and national population density, mainly for *Erinaceus* species. These include spotlight surveys [15,16,17,18], footprint tunnel surveys [19,20,21,22], citizen science surveys [23,24,25], game bag surveys [26] and roadkill surveys [27]. These surveys are often used to assess occupancy rather than population density and demography. There has been a sparsity of long term demographic studies in all hedgehog species. There are some valuable capture-mark-recapture studies [28,29,30] that have investigated population dynamics and density of *E. europaeus*, but there are no such studies for other hedgehog species, including *Paraechinus*. Capture-mark-recapture methodology entails capturing and marking individuals then releasing them to re-mix with the local population. Individuals are then recaptured regularly over the study period, giving each individual a capture history. Two types of models can be fitted to these capture histories in order to estimate population size. The first are those for closed populations, where population size is assumed to be constant throughout the study period and there is no emigration or immigration [31,32]. The second is open population models such as Jolly-Seber and Cormack-Jolly-Seber models that can be used to estimate population size and parameters for survival and capture probability in an open population [33,34,35,36].

In this paper, we report, for the first time, the population dynamics of a free-ranging Ethiopian hedgehog population based on a capture-mark-recapture study in Qatar. We present data from a two year study to estimate hedgehog population size, growth rate, capture rate and survivability, in a discrete study area. The study of this population has not only resulted in population census methodology that is transferable to other hedgehog species but also allows for comparison of population density and dynamics with that of the better studied European hedgehog.

## 2. Materials and Methods

### 2.1. Study Area and Animal Capture

The study area consisted of ~15 km^2^ of arid land around the Qatar University Farm (25°48′ N, 51°20′ E) in northern Qatar. The area included 11 active farms that were irrigated daily using underground water extracted through deep wells. Except for those farms, the area was an arid plain with a total annual precipitation of less than 100 mm, and the surface was predominantly covered by desert pavement with exposed loose gravels. The ambient air temperature ranges between ~5 °C in the early morning in winter and ~50 °C in the early afternoon in summer. There was little vegetation except for isolated short acacia tress and ephemeral grass patches emerging after rains in cold months. Various structures created by human activities, such as rubbish dumps, piles of abandoned building materials, and soil mounds, were ubiquitously found across the study area. Fieldwork was carried out between April 2010 and April 2012.

**Figure 1:**
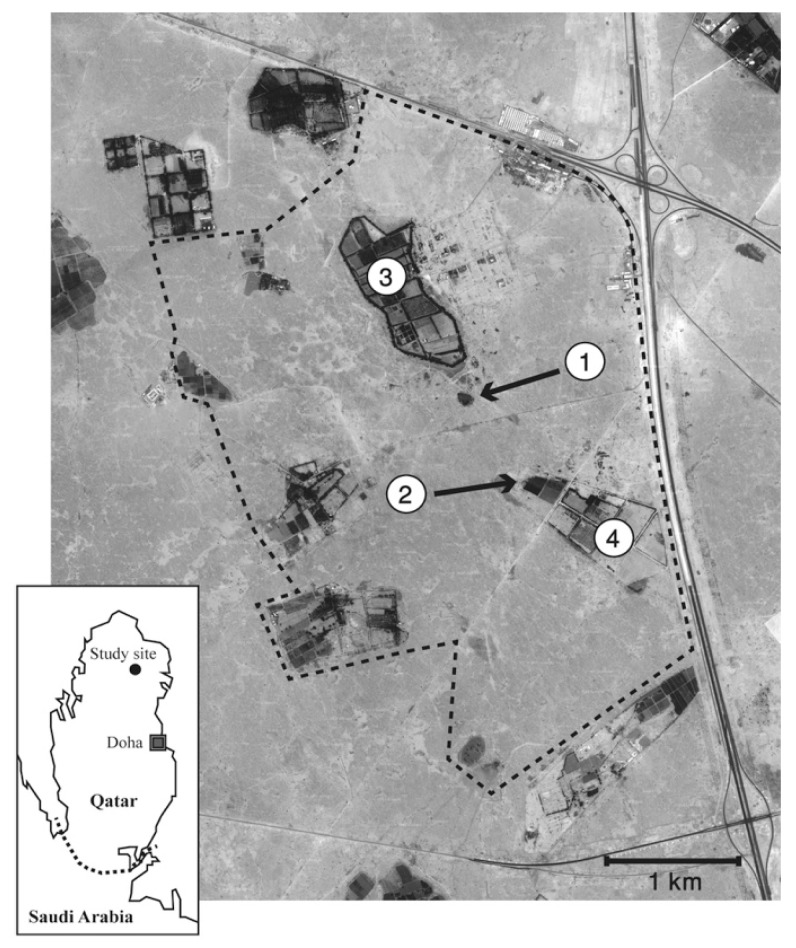
Map of the 15 km^2^ study site in Qatar where Ethiopian hedgehogs were captured by hand as part of a monthly capture-recapture experiment from April 2010 to April 2012 (GoogleEarth Image Copyright 2018 DigitalGlobe). The dashed line indicates an 8.5 km^2^ focal search area. Numbers indicate: **①**: The “Rubbish Mound” where a higher concentration of hedgehogs was found throughout the study probably due to year-round availability of food resources; **②**: “Municipal Farm” where permanent grass fields attracted hedgehogs; **③**: Rawdat Al-Faras Research Station where street lights across the farm increased the chance of locating hedgehogs; **④**: Qatar University Farm where the field station was located.

A consecutive four-night hedgehog capture survey was conducted, from dusk until dawn, once a month in an area of ~8.5 km^2^ (regular survey area) by a field team of 1–3 individuals. Hedgehogs were captured by hand, usually curling into a ball, and were processed at the capture sites without anesthesia or sedation. Hedgehogs were individually marked by painting the spines with unique combinations of nail polishes of different colors, and sexed before they were released. A hedgehog was classified as a juvenile if an animal was less than six months old or if it was a new individual and weighed less than 200 g. Each hedgehog was only processed once during the four night survey. A substantial amount of capturing efforts was made (1) around the “Rubbish Mound” (Figure 1, location **①**) where a higher concentration of hedgehogs was found throughout the year probably due to year-round availability of food resources (although the rubbish mound was partially cleared in March 2011, and a further major cleaning operation started in March 2012); (2) “Municipal Farm” (Figure 1, location **②**) where permanent grass fields seemed to produce rich invertebrate communities seasonally; (3) “Rawdat Al Faras Farm” (Figure 1, location **③**); and (4) Qatar University Farm where the field station was located (Figure 1, location **④**). In addition to captures at those sites, hedgehogs were captured wherever and whenever they were found in the regular survey area. Searching was carried out as follows: firstly each farm and the rubbish mound were searched because hedgehogs tended to nest in these areas and could be captured as they emerged from their nests. Each farm was searched once and the rubbish mound twice during this time, finishing around 22:00. The rest of the study area was then searched from the north to the south, with the aim of randomly encountering hedgehogs. This area was completely searched over the four days but the whole area was not covered every evening. We divided the year into four hedgehog seasons as follows; Early Breeding Season; February–April, Late Breeding Season; May–July, Autumn Season; August–October and Winter Season; November–January [5].

### 2.2. Data Analysis

The statistical analysis of the capture-recapture data was carried out in the R package “RCapture” (R Core Team 2014), following the paper ‘Rcapture: Loglinear Models for Capture-Recapture in R′ by Baillargeon and Rivest [37]. This package cannot handle irregular capture intervals, and because some months of captures were missed in early 2010, we decided to subset the data into two blocks; all continuous months in the study (19 months from October 2010 to April 2012) and excluding the Winter Season (nine months from February 2011 to October 2011). Analyzing the data excluding the Winter Season allowed us to look at population demographics over one year of hedgehog activity, where the capture rate is not affected by the change of behavior that this hedgehog species (and other hedgehog species) exhibit over winter [3,6,11]. The analysis was also run separately for males and females. We also ran separate analyses for hedgehogs that were juveniles at first capture versus those deemed to be adults at first capture. The analyses on these different age cohorts were only performed on one year of data excluding winter months (2011) because over more than one year those hedgehogs deemed juveniles or at first capture would have become adults during the course of the study. However, the juvenile category does include those deemed a juvenile at first capture in 2010, i.e., by summer 2011 they would be subadults that have overwintered once. Therefore, in these analyses juveniles and these subadults were combined into one group and are hereafter called juveniles.

The RCapture package fits both open (Jolly-Seber and Cormack-Jolly-Seber) and closed population models to estimate N (the population size) along with parameters for capture probability at each sampling occasion, and survival and the number of new entrants between sampling occasions [37]. The study area was not a closed population and therefore the open models are most likely suitable for this data. However, we did some exploratory analysis using both closed and open models to confirm which fit the data best. Model fit was judged on Akaike Information Criterion (AIC) values, the lowest AIC being deemed as the best fitting model. Following Baillargeon and Rivest 2007 [37], we also examined heterogeneity plots of the capture histories, plotted the Pearson residuals from each model, and performed tests of model fit. If Pearson residuals were high or there appeared to be heterogeneity in the data then models were adjusted in a number of ways, for example, the model was re-run with capture histories with high residuals removed [37]. We also checked whether individuals captured at all sampling occasions or at only one may have had a big influence on the model fit. The output from the open population models in the Rcapture package includes a test for trap effect. The AIC value for the model including the trap effect was compared with that for a homogenous trap effect to investigate whether there was a difference in capture probability through time because of a behavioral response to capture. Finally, we ran the same models with equal capture probabilities defined and compared their fit to all of the models.

After selecting the best fitting models for each subset of our data, we obtained the total population estimate along with monthly population size, survival and capture rate for each block of data analyzed. We then tested potential differences in these estimates between males and females and adults and juveniles by constructing a series of linear models in R (R Core Team, Vienna, Austria, 2014). We examined the residuals of these linear models for normality and transformed the dependent where appropriate. Density was calculated based on the mean monthly N over the 8.5 km^2^ regular search area. All means are presented as mean ± the standard error of the mean.

## 3. Results

We recorded 1190 captures between April 2010 and April 2012. Males were highly statistically significantly more likely to be captured (744 times) in comparison to females (427 times) (binominal test: *p* < 0.001), although the overall sex ratio of captured animals did not statistically significantly deviate from 1:1 (87 males and 74 females, binominal test: *p* = 0.34). Based on monthly data larger numbers of males were recorded than those of females throughout the study period. This bias towards male capture was statistically significantly more obvious during the Winter Season (ANOVA, F_3_ = 6.6, *p* = 0.003) whilst there was no significant difference amongst the other hedgehog seasons (ANOVA, F_2_ = 0.52, *p* = 0.61). This seasonal difference might be related to a statistically significantly smaller average number of female hedgehogs caught during the Winter Season (ANOVA, F_3_ = 5.3, *p* = 0.008), whilst there was no such seasonality detected in males (ANOVA, F_3_ = 2.2, *p* = 0.12). The foregoing results may suggest that the catchability of males was higher than females, and also female catchability decreased in winter. Therefore, this finding supports our decision to include some population estimates of males and females separately, in order to distinguish different patterns in male and female demography, and also to perform a separate analysis with winter excluded.

### 3.1. Model Fit

**Figure 2:**
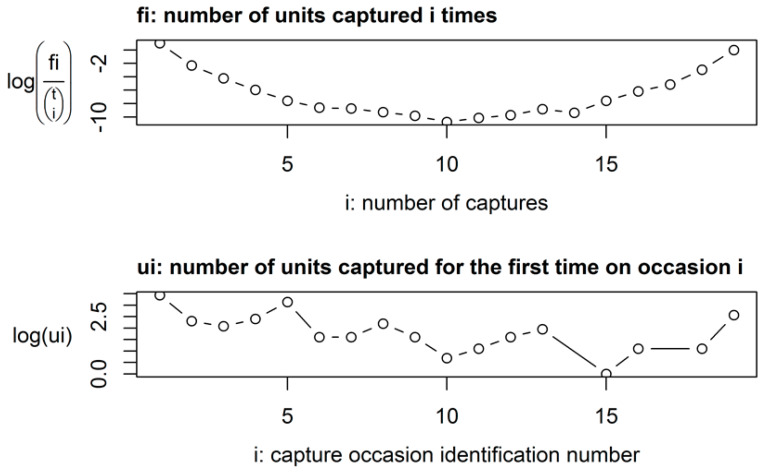
Exploratory heterogeneity graph showing descriptive data from the capture histories of Ethiopian hedgehogs caught as part of a capture-mark-recapture study in Qatar over 19 months (October 2010 to April 2012).

As predicted, open population models fit the data better than closed population models. In some cases exploratory plots of heterogeneity were u-shaped rather than linear; therefore showing that heterogeneity in the capture probabilities may be an issue (e.g., Figure 2). We were able to improve model fit by adjusting the models, for example removing those animals captured on every occasion to reduce heterogeneity in the data. We also removed capture histories where plots of residuals revealed very large residuals. The best-fitting models and the adjustments made to them are presented in Table 1, alongside the models with no modifications. These adjustments did not dramatically alter population estimates but did improve the standard error of the population estimates and the model fit (Table 1). In all cases, the *p*-value of goodness of fit testing, based on the deviance of the models, was >0.05, indicating that our models adequately fit our data.

### 3.2. Population Size

Our models resulted in a range of population estimates for our study site (Table 1). If we are to include all 19 continuous months and hedgehogs caught during this period (144 hedgehogs) then we estimate a mean monthly population of 60 ± 2.9 hedgehogs on our study site. If we exclude the Winter Season (112 hedgehogs included), the number of hedgehogs is slightly smaller at 54 ± 3.7 hedgehogs. There was no statistically significant difference between the monthly abundance estimates from these two time periods (ANOVA, F_1,22_ = 1.67, *p* = 0.21). These estimates give a density of 7 hedgehogs per km^2^ in our 8.5 km^2^ focal search area, or a density of 6.3 hedgehogs per km^2^ excluding winter. As expected from observations on the ground, the estimated population size was larger for males than females in all our models (Table 1). 

Twenty-two percent of hedgehogs were caught on the very first sampling occasion. This figure was higher for males (24%) than it was for females (21%). This pattern was followed when excluding the Winter Season, with 63% of male hedgehogs captured in the study caught on the first occasion compared with 53% of females. Only 4.8% of hedgehogs caught at the very first sampling occasion were not captured again throughout the study. The figure was 12.8% for the analysis performed excluding the Winter Season.

**Figure 3:**
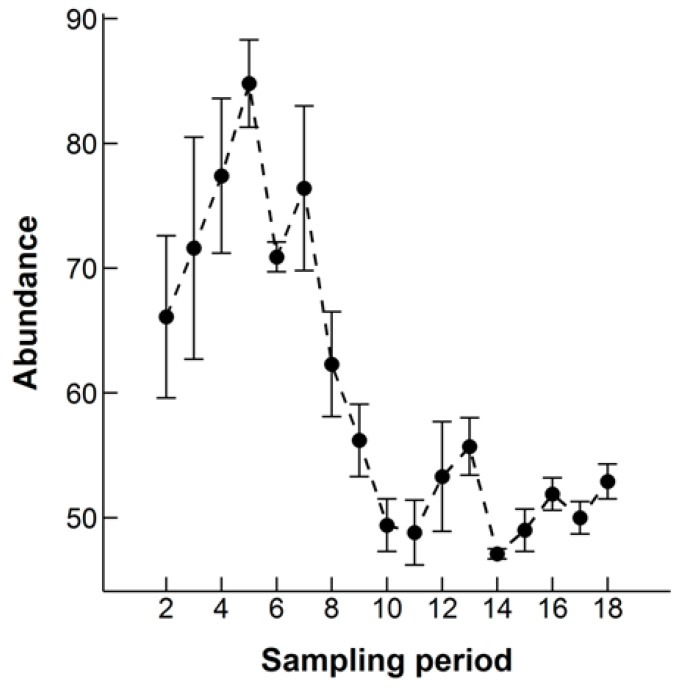
The estimated abundance of Ethiopian hedgehogs in an 8.5 km^2^ search area in Qatar at each monthly sampling occasion over a 19 month period (October 2010 to April 2012). Bars indicate the standard error of each abundance estimate.

**Figure 4:**
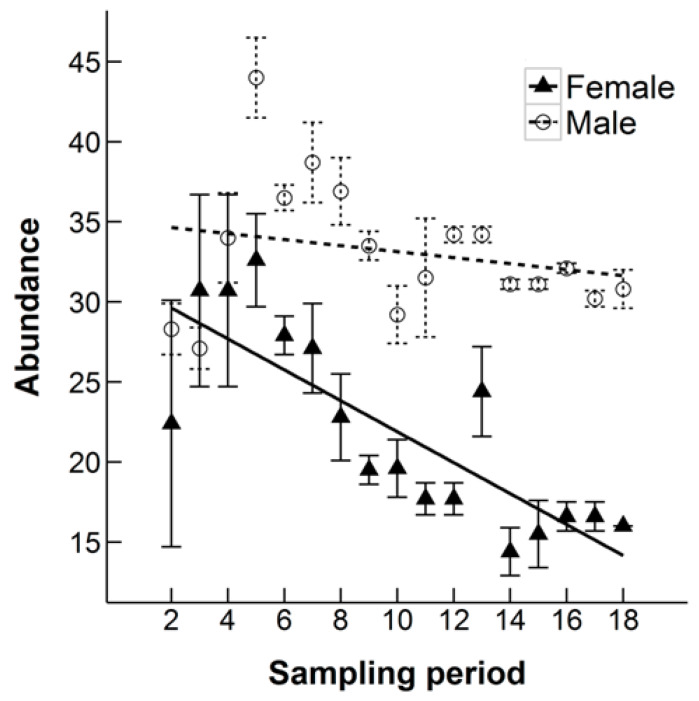
The estimated abundance of male and female Ethiopian hedgehogs in an 8.5 km^2^ search area in Qatar at each monthly sampling occasion over a 19 month period (October 2010 to April 2012). Error bars shown are the standard error of the estimate. Linear regression lines are also displayed for each sex.

**Figure 5:**
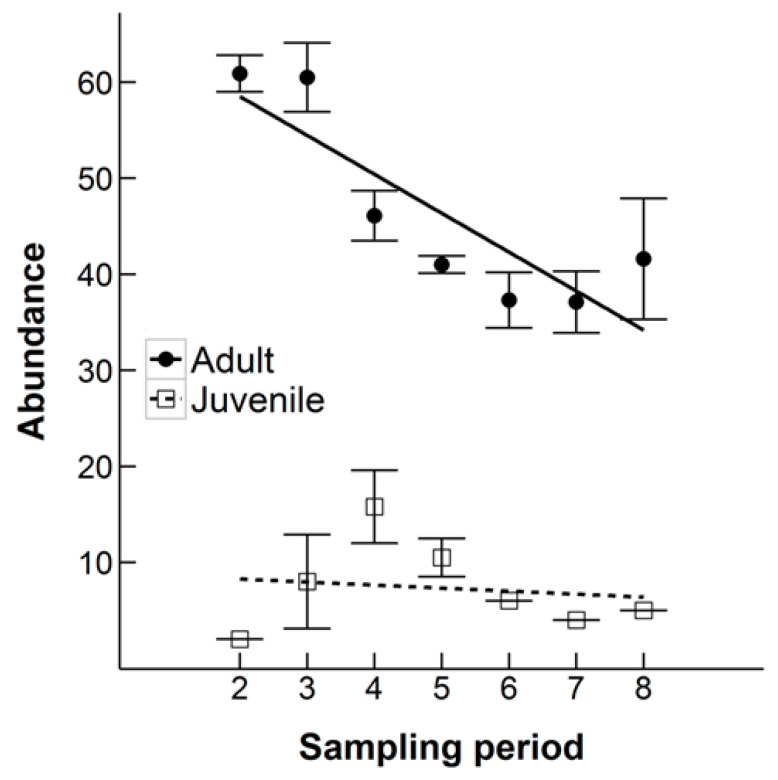
The estimated abundance of adult and juvenile (including subadult) Ethiopian hedgehogs in an 8.5 km^2^ search area in Qatar at each monthly sampling occasion over a nine-month period (February 2011-October 2011). Linear regression lines are displayed for each group, error bars are the standard error of the monthly abundance estimate.

The monthly estimation of N at each sampling occasion decreased throughout both time periods analyzed (all 19 months: ANOVA, F_1,10_ = 17.78, *p* = 0.0002, excluding the Winter Season: ANOVA, F_1,10_ = 33.68, *p* = 0.0002), suggesting that the population size was decreasing at the study site (Figure 3). Over the full 19 months, the monthly population estimate fell by 20%. The average monthly growth rate was −0.86%. The abundance of males at each sampling occasion was significantly higher over both analyzed time periods (all 19 months: ANOVA, F_1,10_ = 70.40, *p* < 0.0001, excluding the Winter Season: ANOVA, F_1,10_ = 75.64, *p* < 0.0001). When all 19 months were included, there was a significant interaction between sex and sampling period (ANOVA, F_1,30_ = 8.08, *p* = 0.008), with a steeper decline in the estimated number of females each month over the course of the study (Figure 4). On average there were estimated to be over seven times more adults (46.36 ± 3.87) than juveniles (7.33 ± 1.75) when excluding the Winter Season, which was highly statistically significant (ANOVA, F_1,10_ = 179.67, *p* < 0.0001, Figure 5). There was a statistically significant interaction between age and sampling occasion (ANOVA, F_1,10_ = 6.60, *p* = 0.03). The estimated number of juveniles and subadults increased during the first four sampling periods then leveled off, whereas the estimated number of adults decreased (Figure 5).

### 3.3. Capture Probability

**Figure 6:**
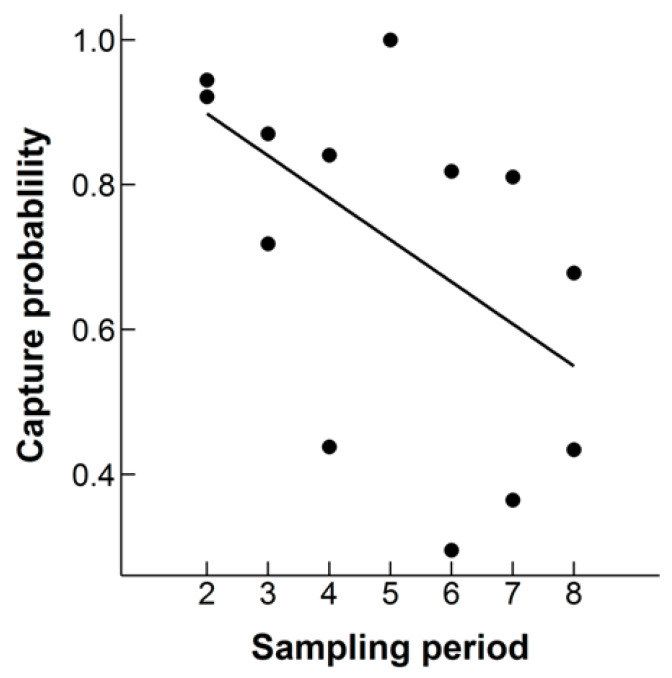
The estimated capture probability of Ethiopian hedgehogs sampled monthly from February 2011 to October 2011 in an 8.5 km^2^ search area in Qatar. A linear regression line is displayed.

Mean capture probability was higher for males (0.70 ± 0.04) than females (0.60 ± 0.06) but this difference was not statistically significant (ANOVA, F_1,30_ = 2.06, *p* = 0.16). Surprisingly, when excluding the Winter Season, the mean capture probability from the best fitting models was higher for females (0.81 ± 0.07) than males (0.63 ± 0.11), however, the difference was also not statistically significant (ANOVA, F_1,10_ = 2.40, *p* = 0.15). Looking at the whole study, there was no effect of sampling period on the capture rate (ANOVA, F_1,30_ = 1.03, *p* = 0.32). However, it may be more appropriate to look at this over the awake period for hedgehogs, indeed there was a near significant effect of sampling period on capture probability when excluding the Winter Season (ANOVA, F_1,10_ = 4.02, *p* = 0.07). Capture probability decreased through time (Figure 6). There was a higher probability of catching juveniles (0.80 ± 0.097) than adults (0.70 ± 0.07), but this was not statistically significant (ANOVA, F_1,10_ = 0.85, *p* = 0.38). Furthermore, there was a near statistically significant interaction between age (adults versus juveniles) and sampling period over this awake period (ANOVA, F_1,10_ = 7.46, *p* = 0.066). Capture probability of adults declined over the awake period, whereas juveniles increased. We also tested to see if there was a trap effect over the course of the study. The AIC value for the models including trap effect was higher than that for a homogenous trap effect in all our models, indicating that any differences in capture probability through time were not down to a behavioral response to capture.

### 3.4. Survival

**Figure 7:**
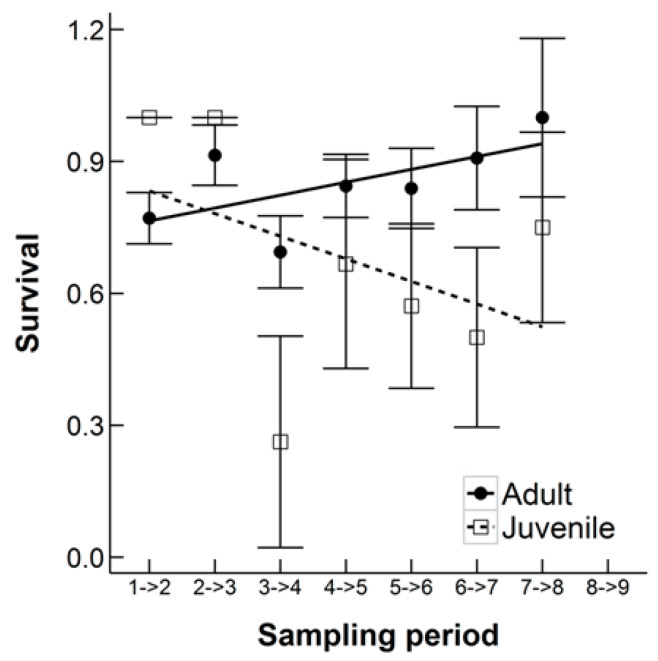
The estimated survival rate of Ethiopian hedgehogs between monthly samples from February 2011 to October 2011 in an 8.5 km^2^ search area in Qatar, showing the survival of adults versus juveniles (including subadults). Linear regression lines are shown for each group, error bars are the standard error of the monthly survival estimate.

Mean survival between sampling occasions was similar for males (0.89 ± 0.02) and females (0.88 ± 0.04). When the Winter Season was excluded, the mean survival between sampling periods was higher for males (0.81 ± 0.05) than females (0.74 ± 0.06). However, this was not statistically significant (ANOVA, F_1,10_ = 0.60, *p* = 0.46). There was also no difference in survival between sampling periods both including and excluding the Winter Season (all 19 months: ANOVA, F_1,30_ = 2.14, *p* = 0.15, excluding winter: ANOVA, F_1,10_ = 0.31, *p* = 0.59). Mean adult survival between monthly captures (0.85 ± 0.04) was greater than mean juvenile survival (0.68 ± 0.10) but this was not statistically significant (ANOVA, F_1,10_ = 0.77, *p* = 0.40). However, there was again a statistically significant interaction between age and sampling period (ANOVA, F_1,10_ = 9.51, *p* = 0.01). Juvenile survival decreased over time whereas adult survival increased (Figure 7), although note there is an outlier in the juvenile estimates and the standard error bars are very large. There were only a few cases where we were able to confidently identify causes of mortality during the study. The main causes were traffic accidents and starvation/exhaustion.

### 3.5. New Entrants

**Figure 8:**
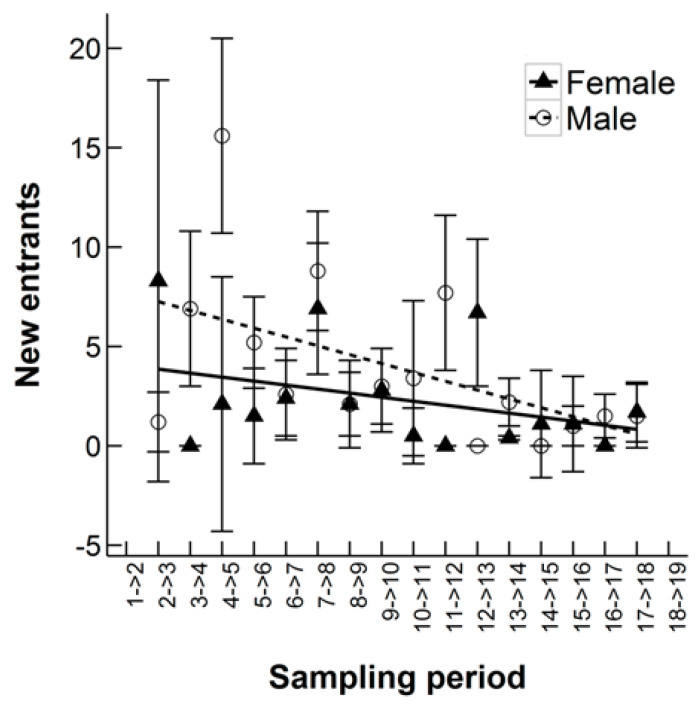
The estimated number of new entrants to a population of Ethiopian hedgehogs between monthly samples from October 2010 to April 2012 in an 8.5 km^2^ search area in Qatar, showing the estimates for females versus males with a linear regression line for each group.

The number of new entrants decreased over time and this was statistically significant when including all 19 sampling periods (ANOVA, F_1,28_ = 7.22, *p* = 0.01, Figure 8). This finding again supports that the population size on the site was decreasing. The mean rate of new arrivals to the study site between sampling occasions was higher for males (all 19 months: 3.92 ± 1.03, excluding the Winter Season: 4.83 ± 0.99) than females (all 19 months: 2.35 ± 0.66, excluding the Winter Season: 3.38 ± 1.1) over both sampling periods, but again this was not statistically significant (all 19 months: ANOVA, F_1,28_ = 2.06, *p* = 0.16, excluding the Winter Season: ANOVA, F_1,8_ = 0.97, *p* = 0.35, Figure 8). The mean number of new entrants between monthly sampling occasions was similar for adults (3.63 ± 0.43) and juveniles (3.47± 1.98). However, note the high standard error for the mean rate of juvenile new entrants.

### 3.6. Evaluation of Methodology

When marking hedgehogs, we found that black, yellow, and non-metallic green nail polish tended to disappear soon, whilst white, red, metallic green, and blue lasted for longer (some even lasted for more than six months). We found that applying the nail polish along the entire length of the spines was more successful because the colors tended to wear off towards the tip of the spines. 

According to our population estimates, we captured between 91.3% and 100% of the local hedgehog population at our study site (Table 2). 

## 4. Discussion

Our results indicate a mean population of 60 hedgehogs at the study site at one time over a 19 month period. The outputs from our models suggest that the monthly population estimate declined over the study period. Survival appeared to be stable throughout the study, potentially a result of a lack of predators of hedgehogs in Qatar [11] and plentiful food resources provided by the rubbish mound and irrigated farms at the site. The rubbish mound was partially cleared in March 2011 (month six of the study), and a further major cleaning operation started in March 2012, potentially resulting in a reduction of resources. Indeed the large drop in abundance was during months seven to ten of the study (Figure 3) and coincides with this change at the study site. However, it was still observed to be the activity center of quite a few animals until it was totally cleared. There were many more adults in the population than juveniles and juvenile survival slightly decreased over time, which could indicate that juvenile survival could be a potential reason for the decrease in monthly abundance estimates. However, there were large standard errors for the estimates of juvenile survival so this result must be interpreted with caution. This decline in juvenile survival could also potentially be down to individuals emigrating from the site as resources at the rubbish mound were reduced. The finding of a population decline is also supported by a statistically significant decline in new entrants (births and/or immigrants) to the population over the study period. The mean number of new entrants between sampling occasions was similar for adults and juveniles. Over one year numbers of juveniles increased initially and then levelled off, as would be expected after breeding activity first peaks in March [5]. However, Ethiopian hedgehogs are thought to have two litters [5] so we would expect to see a second peak in the abundance of juveniles later on in the season and we did not, suggesting the success of the second breeding attempt could be an issue for population recruitment. Conversely, the expected second peak is substantially smaller than the first one [5], and potentially this was not picked up in our models.

Survival in *E. europaeus* has been shown to be impacted by a high predation rate [38], road casualties [29,39], and poor survival over winter, particularly for juveniles [29,40]. There were only a few cases where we were able to identify mortality and its cause during the study and these included traffic accidents and starvation/exhaustion, which we suggest is a result of searching for mates in males and rearing young in females. In all 19 months of our study (including two winters), the estimated survival between monthly sampling occasions ranged from 38.6% to 100% (mean 75%). When excluding the Winter Season, the mean survival between sampling periods was higher ranging from 66% to 100% (mean 84%). These results indicate mortality over winter, may be an important factor in survival in the population studied here.

The reduction in new entrants over the study period could also be a result of a lack of immigration to the population. Only 4.8% of hedgehogs caught at the very first sampling occasion were not captured again throughout the study, indicating that there was not a high number of transients at the site and therefore not much immigration and emigration. The area surrounding the study site consisted of similar habitat with irrigated farms surrounded by arid desert. Released Ethiopian hedgehogs have been shown to travel 131.4–426.7 m per evening and utilize more than one irrigated farm [41], so dispersal between farms is likely and we would expect some immigration from outside of the study area. However, we can assume that distances of greater than the 8.5 km^2^ search area would be much less likely and although wild individuals of this species may have home ranges of up to 230 ha [6,13], their home ranges have been shown to be smaller in resource-rich habitats, such as around irrigated farms [13]. It may be that the species is reasonably site faithful where resources are plentiful and radio-tracking studies at the same study site found that home ranges centered around the rubbish dump and irrigated farms, and hedgehogs did not appear to leave the 15 km^2^ study site [6].

There was a higher abundance of males at the study site, which has been found for populations of *Erinaceus* species e.g., References [1,38,42]. This finding could be down to the higher capture probability of males because, like Erinaceous hedgehogs, they tend to range further distances than females [6]. This idea is supported by the sex ratio of 1:1 in raw captures. However, it could also be due to the decline in estimated monthly abundance being steeper for females than males. There were not enough juveniles in the dataset to investigate whether survival was lower for female juveniles than male juveniles at the site.

Another potential factor in the population decline is that, when the Winter Season was excluded, capture probability decreased throughout the year. This finding could be down to the reduction of the population size but we must also consider that it somehow became harder to capture hedgehogs; perhaps they began to avoid the site to evade being captured, or became ‘trap happy’. The AIC values for the models including trap effect was higher than the AIC values for the same models with a homogenous trap effect included, indicating that there was not a difference in capture probability through time because of a behavioral response to capture. Another potential reason for this is that hedgehog activity peaked in the early breeding season, resulting in less hedgehog movement later in the season and thus fewer captures [5,6]. Capture probability of juveniles increased over one season, likely because as the hedgehogs begin to breed after emergence from torpor, juveniles enter the population and more will be captured as they come out of the nest(s) and start to move around the site.

Studies of hedgehog demography over longer periods have shown that big fluctuations in population size are common. Kristiansson studied a population of *E. europaeus* in Sweden over eight years and found the population was in decline for the first three years, increased for the next three years and then declined again [29,43,44]. Akin to our study, a decreasing population was linked to low numbers of juveniles. Again, one potential factor in the survival of juveniles indicated in the study was survival over winter, especially with respect to colder winters. Although winters are not particularly cold at our study site and some hedgehogs, particularly males, remain active to some degree over winter [6], hedgehogs do enter torpor for short periods [3]. To ascertain whether the population on our study site is in long term decline or this is merely a fluctuation in population size, a much longer-term study is needed, including observations on causes of mortality in different age classes, principally over winter.

### 4.1. Comparison with Other Hedgehog Species

Using the population estimate from all months of the study, the density of hedgehogs in the regular search area was 7 hedgehogs per km^2^. Comparative estimates of European hedgehog density vary greatly with habitat type and methodology. For example, Hubert et al. 2011 [45] found that mean hedgehog density, was 4.4 individuals per km^2^ in rural areas of France and 36.5 individuals per km^2^ in urban areas. Young et al. 2006 [46] found a mean density of nine individuals per km^2^ in a survey of pasture fields in England, the same survey in amenity grassland found up to 154 hedgehogs per km^2^. In their capture-mark-recapture study, Reeve 1981 found a population size of 82.5 hedgehogs per km^2^ on a suburban golf course in England. Jackson et al. 2007 [18] studied the abundance of introduced hedgehogs on the Scottish island of South Uist (where hedgehogs are thriving) and found 31.8 hedgehogs per km^2^ in sandy-soiled flat dune grassland habitat and 15.4 peaty-soiled pastureland. It seems the density found in this study is fairly low compared to that of Erinaceus species in a reasonably productive habitat, which may be expected given the nature of a less productive hyper-arid environment at the study site. This idea is supported by the finding that Ethiopian hedgehogs in Qatar have been shown to have a larger home range than their European counterparts, in spite of the latter being substantially heavier, likely because of these dispersed resources [6]. The density estimate presented here may also be inflated because of the artificial food sources at the site and it is likely in areas of ‘natural desert’ in Qatar the hedgehog density is lower.

Survival rates also vary between studies of *E. europaeus* and we must be careful about drawing conclusions from these comparisons as figures are presented from a range of habitats and methodologies. Translocated and released hedgehogs have a survival rate of between 40% and 77% after several weeks in the wild [47,48,49,50]. Survival of individuals in extant populations over short periods is higher, for example nearly 95% during an eight week study in urban habitats in the UK [49]. Reeve 1981 [28] found a survival rate of 62%, over one year including winter, however over two winters this was reduced to 37%. Over the whole of our study, including two winters, the mean monthly survival rate between sampling occasions was higher at 75%. Kristiansson 1990 [29] found an average annual survival of 66% in juveniles and 55% in adults. Whereas we found a similar mean monthly survival rate of 64% for juveniles and subadults over nine months, our figure for adults was much higher at 85%. Rasmussen et al. 2019 [51] found a slightly higher juvenile survival rate of 70% over one year, with an over winter survival rate of 89%, they attribute this high winter survival to the suburban habitat type. 

### 4.2. Evaluation of Methodology

Our results suggest that we were able to capture most of the local population and therefore we conclude that sampling at key habitats with spotlights for hedgehogs seems to be sufficient in capturing most of the hedgehog population at a given site. Other capture-mark-recapture experiments on *E. europaeus* have also found that they were also efficiently able to catch a high proportion of the estimated population size [28,29]. Like the hedgehogs in this study that were attracted to the ‘rubbish mound’, European hedgehogs may also be attracted to areas of abundant food e.g., urban habitats with plentiful pet food [42,45,52], and we conclude that sampling in these areas may be sufficient to gain knowledge of the local hedgehog population. However, we must take note that our density estimate cannot be extrapolated over the whole 15 km^2^ study site because we cannot assume the rest of the habitat at the study area to be of the same quality as our focal search area, and likewise we cannot assume all hedgehogs in the 15 km^2^ area were attracted to our key survey areas. Further study could be conducted by carrying out a mark-recapture-study on the less utilized arid areas of the study site. Additionally, it would be interesting for further study to compare the density found here with areas of ‘natural’ desert in Qatar where anthropogenic intervention to the habitat is minimal. 

Jolly-Seber and Cormack-Jolly-Seber modelling may underestimate the population size at a study site, especially in short-lived species [53]. However, hedgehogs were observed to live multiple years during the study with some animals first encountered as adults in April 2010 still alive when the project finished in April 2012, which may reduce the risk of us underestimating the population. However, a future study could include performing a “robust design” analysis, whereby hedgehogs would be processed multiple times during the four-day survey and two levels of modeling are carried out; closed population modeling between each consecutive night of the survey and open models between each monthly visit [37,53,54]. Robust design methodology is less likely to underestimate the population size [53] and would make an interesting comparison with the modeling presented here.

We were able to sufficiently identify the marked hedgehogs in the study using colored nail polish, but this method requires re-application. We found that Since our study improved ways of marking hedgehogs, such as using numbered plastic tubing, have been successfully tested and could be used to increase confidence in animal identification in future studies [55].

## 5. Conclusions

We successfully used capture-mark-recapture methodology to come up with a range of population estimates for the Ethiopian hedgehog. We found that the estimated monthly population at our study site had decreased over the 19 months of our study and potential causes of this include poor juvenile survival and a lack of immigration in to the study area. However, a longer term study is needed to ascertain if this is a sustained population decline and to confirm the causes. As well as obtaining a range of population estimates, our methodology allowed us to report a range of valuable demographic parameters that give the first insight into the population dynamics of Ethiopian hedgehogs in Qatar. The methods presented here are transferrable to other hedgehog species in a range of habitats.

## Figures and Tables

**Table 1:** Estimated population size of Ethiopian hedgehogs at a study site in Qatar from a series of open population models constructed in the R package RCapture. Results are presented from the models with no modifications and the best fitting models, determined by AIC values.

Subset	Block	No. in Model ^1^	Adjustments to Improve Model Fit	AIC	N_tot_^2^	SE	N ^3^	SE	Density (km^−2^) ^4^
All	All 19 months	144	None	1180	149	2.7	55	2.4	6.5
All	All 19 months	144	Excluding those captured all 19 times and including residuals <10	439	151	0	60	2.9	7.0
Males	All 19 months	75	None	787	77	1.6	32	1.1	3.8
Males	All 19 months	75	Excluding those captured all 19 times and including residuals <50	489	77	0	33	1.0	3.9
Females	All 19 months	62	None	540	65	2.3	22	1.5	2.6
Females	All 19 months	62	Capture constant on model and including residuals <800 ^5^	511	65	1.9	21	1.5	2.5
All	Excluding winter	112	None	346	115	2.3	52	3.8	6.1
All	Excluding winter	112	Excluding those captured all 9 times	327	117	1.9	54	3.7	6.3
Males	Excluding winter	59	None	244	60	1.2	30	1.9	3.5
Males	Excluding winter	59	Excluding those captured 8 or 9 times and including residuals <6	177	61	0	30	1.7	3.5
Females	Excluding winter	47	None	180	47	1.1	19	1.9	2.2
Females	Excluding winter	47	Excluding those captured 8 or 9 times	142	47	0	19	1.9	2.2
Adults	Excluding winter	92	None	304	93	1.5	45	3.9	5.3
Adults	Excluding winter	92	Excluding those caught all 9 times and including residuals <10	266	94	0	46	3.9	5.4
Juveniles	Excluding winter	21	None	100	26	5.1	7	1.8	0.8 ^6^

^1^ The number of hedgehogs captured in this time period and included in the model—note, not all hedgehogs could be aged and/or sexed hence males and females do not add up to the total number of hedgehogs captured. ^2^ The total number of hedgehogs estimated to be on site during the period analyzed. ^3^ The mean monthly number of hedgehogs estimated to be on the site during the period analyzed. ^4^ Density was calculated based on the mean monthly N over the 8.5 km^2^ regular search area. ^5^ Note this model had some extremely high residuals but the lowest number at which the model would converge was <800. ^6^ The null model was deemed the best fitting model for juveniles.

**Table 2:** Percentage of a hedgehog population estimated from Jolly-Seber modeling in the R package Rcapture that was captured in the field.

Subset	Block	No. Hedgehogs ^1^	N_tot_	% of Population Captured
All	All 19 months	144	151	95.36
Males	All 19 months	75	77	97.40
Females	All 19 months	62	65	95.38
All	Excluding winter	112	117	95.73
Males	Excluding winter	59	61	96.72
Females	Excluding winter	47	47	100.00
Adults	Excluding winter	92	94	97.87
Juveniles	Excluding winter	21	23	91.30

^1^ The number of hedgehogs captured in this time period and included in the model—note, not all hedgehogs could be aged and/or sexed hence males and females do not add up to the total number of hedgehogs captured.
